# Shifting Epidemiology of *Stenotrophomonas maltophilia*: Specimen-, Ward-, and Time-Dependent Patterns in a Tertiary Hospital 2020–2025—An Observational Study

**DOI:** 10.3390/jcm15145636

**Published:** 2026-07-17

**Authors:** Aneta Guzek, Anna Olczak-Pieńkowska, Wiesław Piechota, Zbigniew Rybicki, Monika Konior, Katarzyna Mackiewicz, Monika Kania, Karol Warda, Dariusz Tomaszewski

**Affiliations:** 1 Department of Laboratory Diagnostics, Section of Microbiology, Military Institute of Medicine–National Research Institute, 04-141 Warsaw, Poland; aguzek@wim.mil.pl (A.G.); kmackiewicz@wim.mil.pl (K.M.); 2 Department of Public Health, Epidemiology and Vaccinology, Military Institute of Medicine–National Research Institute, 04-141 Warsaw, Poland; aolczak-pienkowska@wim.mil.pl (A.O.-P.); mkania1@wim.mil.pl (M.K.); 3 Department of Laboratory Diagnostics, Military Institute of Medicine–National Research Institute, 04-141 Warsaw, Poland; wpiechota@wim.mil.pl; 4 Department of Anesthesiology and Intensive Therapy, Military Institute of Medicine–National Research Institute, 04-141 Warsaw, Poland; zrybicki@wim.mil.pl; 5 Department of Epidemiology and Tropical Medicine, Military Institute of Medicine–National Research Institute, 04-141 Warsaw, Poland; mkonior@wim.mil.pl; 6 Department of Clinical Epidemiology and Hospital Infection Control, Military Institute of Medicine–National Research Institute, 04-141 Warsaw, Poland; kwarda@wim.mil.pl; 7 Department of Anesthesiology and Intensive Therapy, Military Institute of Aviation Medicine, 01-755 Warsaw, Poland

**Keywords:** *Stenotrophomonas maltophilia*, antimicrobial resistance, epidemiology, non-fermenting Gram-negative bacilli, hospital surveillance, EUCAST

## Abstract

**Background/Objectives**: *Stenotrophomonas maltophilia* is a critical opportunistic pathogen, yet its long-term epidemiology remains underrepresented in global surveillance frameworks like WHO GLASS. We aimed to analyze its six-year epidemiological trend in a large Polish tertiary hospital. **Methods**: A retrospective study (2020–2025) of 399 non-duplicate isolates was conducted. Identification was performed using MALDI-TOF MS, and susceptibility was analyzed according to EUCAST breakpoints. Antibiotic consumption (DDD/100 patient-days) was correlated with isolation trends. **Results**: Wound/skin (36.8%) and lower respiratory tract (35.8%) samples predominated. A significant rise in respiratory isolates was observed post-2020, coinciding with peak carbapenem consumption in ICU and surgical wards. Ward-level analysis demonstrated that intensive care unit status (β = 68.9, 95% CI 46.1–91.8, *p* < 0.001) and carbapenem consumption (β = 0.080, 95% CI 0.004–0.156, *p* = 0.041) were independently associated with *S. maltophilia* isolation burden after adjustment for ward type (adjusted R^2^ = 0.80). While trimethoprim–sulfamethoxazole remained highly active, levofloxacin susceptibility showed high interannual variability. **Conclusions**: *S. maltophilia* is a stable and clinically significant threat in high-intensity hospital environments. Our findings support the inclusion of this pathogen in international AMR monitoring (GLASS) and emphasize strict carbapenem stewardship to curb its emergence.

## 1. Introduction

*Stenotrophomonas maltophilia* has emerged as an important opportunistic pathogen in healthcare settings, particularly among immunocompromised patients and those undergoing invasive procedures. This Gram-negative, non-fermentative bacillus is characterized by its intrinsic resistance to multiple classes of antimicrobials, including carbapenems, aminoglycosides, and many β-lactams, which significantly limits therapeutic options [1,2]. This organism has been increasingly associated with severe nosocomial infections, such as ventilator-associated pneumonia, bloodstream infections, and urinary tract infections, often in critically ill patients or those with prolonged hospitalization [3,4,5].

Large tertiary-care university hospitals represent an environment with a particularly high risk of *S. maltophilia* colonization and infection. The combination of advanced diagnostic and therapeutic procedures, frequent use of wide-spectrum antibiotics, and the concentration of patients with hematological malignancies, solid organ transplants, or requiring mechanical ventilation contributes to pathogen persistence and clinical relevance [6,7]. Moreover, outbreaks of *S. maltophilia* have been linked to contaminated hospital equipment and water sources, underlining the importance of infection prevention and control strategies.

The study aimed to analyze the epidemiology of *S. maltophilia* isolates recovered from hospitalized patients over six years (2020–2025), with particular emphasis on the distribution by clinical specimen type, hospital department, and temporal trends. Additionally, we evaluated annual antimicrobial susceptibility patterns for key therapeutic agents. We also compared the relative prevalence of *S. maltophilia* with that of other non-fermenting Gram-negative bacilli across major specimen categories.

This study was designed as a laboratory-based ecological surveillance analysis at the ward level, focusing on aggregated microbiological and antimicrobial consumption data rather than individual patient-level clinical outcomes.

## 2. Materials and Methods

### 2.1. Data Collection

The study was designed as a single-center retrospective study. The project was conducted at the Microbiology Laboratory of the Military Institute of Medicine–National Research Institute in Warsaw, Poland. The Military Institute of Medicine is a 1000-bed tertiary-care university hospital and the regional trauma center. Only adult patients were included in the analysis; neonatal and pediatric samples were excluded.

Due to the retrospective laboratory-based design, detailed individual patient-level clinical data (e.g., comorbidities, duration of hospitalization prior to pathogen isolation, mechanical ventilation status, invasive device use, and patients’ mortality) were not available for analysis.

### 2.2. Pathogen Detection

All clinical isolates were identified using Matrix-Assisted Laser Desorption Ionization-Time of Flight mass spectrometry (MALDI-TOF MS; VITEK MS, bioMérieux, Marcy-l’Étoile, France). Among all identified organisms, only non-duplicate *Stenotrophomonas maltophilia* isolates were included in the analysis. Gram-positive cocci and Gram-negative bacilli belonging to the order *Enterobacterales* were excluded.

### 2.3. Antimicrobial Susceptibility Testing

Antimicrobial susceptibility testing was performed using the VITEK 2 automated system (bioMérieux, France) according to the manufacturer’s instructions. The AST-N331 card, designed for Gram-negative non-fermenting bacteria, was used. Susceptibility results were interpreted according to the European Committee on Antimicrobial Susceptibility Testing (EUCAST) criteria available at the time of isolate collection. Not all isolates underwent susceptibility testing each year due to changes in laboratory testing panels, EUCAST breakpoint removal, and occasional sample viability issues.

Due to the intrinsic resistance of *S. maltophilia* to multiple antimicrobial classes, including β-lactams, carbapenems, aminoglycosides, and most quinolones, routine clinical reporting of antimicrobial susceptibility results in 2020–2022 was limited to trimethoprim–sulfamethoxazole, levofloxacin, and ceftazidime, in accordance with the EUCAST recommendations valid at that time.

According to the EUCAST Guidance on antimicrobial agents for *Stenotrophomonas maltophilia* (published on 15 November 2024), EUCAST has withdrawn clinical breakpoints for fluoroquinolones and ceftazidime due to insufficient clinical evidence; therefore, categorical interpretation is currently not recommended. Consequently, interpretation of susceptibility results for these agents against *S. maltophilia* is not recommended due to insufficient clinical and microbiological evidence [8,9]. At present, EUCAST retains clinical breakpoints only for trimethoprim–sulfamethoxazole.

To ensure strict compliance with current EUCAST recommendations, susceptibility results for levofloxacin and ceftazidime are presented for descriptive purposes only and were not subjected to categorical interpretation.

Quality control of susceptibility testing was performed using reference strains of *Pseudomonas aeruginosa* ATCC 27853 and *Escherichia coli* ATCC 25922.

### 2.4. Antibiotic Consumption Analysis

Ward-level antibiotic consumption data were obtained from the hospital pharmacy database. Antibiotic consumption was expressed as defined daily doses (DDD) per 100 patient-days, according to the World Health Organization Anatomical Therapeutic Chemical (ATC) classification system. Systemic antimicrobials (ATC J01) and carbapenems (ATC J01DH) were analyzed annually and stratified by clinical ward for the years 2020–2025.

### 2.5. Statistical Analysis

Categorical data are summarized as frequencies and percentages. Continuous variables are expressed as means with standard deviations or medians with interquartile ranges, as appropriate.

Annual trends in isolate distribution by specimen type and hospital ward were analyzed descriptively. The relative abundance of *S. maltophilia* among non-fermenting Gram-negative bacilli was calculated as a proportion of *S. maltophilia* isolates to the total number of non-fermenters recovered from each specimen type per year.

To explore the relationship between antimicrobial pressure and *S. maltophilia* isolation patterns, we performed an ecological analysis at the ward level. For each of the 16 clinical wards included in the study, we calculated the mean carbapenem consumption (defined daily dose per 100 patient-days, averaged across 2020–2025) and the total number of *S. maltophilia* isolates recovered during the study period.

The bivariate association between carbapenem consumption and isolate burden was initially assessed using the Pearson correlation coefficient. To account for potential confounding by ward type and overall antimicrobial pressure, we subsequently performed multivariable linear regression. The dependent variable was the total number of *S. maltophilia* isolates per ward. Independent variables included mean carbapenem consumption (ATC J01DH), mean total systemic antibiotic consumption (ATC J01), and ward type (intensive care unit vs. non-intensive care unit). Collinearity among predictors was assessed using variance inflation factors (VIFs), with values above 5 indicating potential multicollinearity. Model fit was evaluated using the adjusted R-squared and F-statistic. Regression diagnostics included examination of residual plots, normal probability plots, and leverage statistics to identify influential observations.

To explore whether the association between carbapenem consumption and *S. maltophilia* isolation differed across ward types, we performed a sensitivity analysis restricted to non-intensive care unit wards. This stratified approach allowed us to assess whether the observed ecological relationship was driven primarily by intensive care settings or reflected a broader pattern across diverse clinical environments.

All statistical analyses were performed in the Anaconda Navigator environment (version 2.7.0, Anaconda Inc., Austin, TX, USA) using Jupyter Notebook (version 7.2.2, Project Jupyter, Berkeley, CA, USA). Regression models were fitted using the statsmodels library (version 0.14.0) in Python (version 3.12.8). A two-tailed *p*-value < 0.05 was considered statistically significant. All figures and the graphical abstract were generated in Python using dedicated plotting libraries.

## 3. Results

Between 2020 and 2025, 399 *S. maltophilia* isolates were obtained from various biological materials. The largest proportion of isolates originated from wounds, abscesses, and skin lesions, accounting for 147 cases (36.8%). Samples from the lower respiratory tract were the second most common source, accounting for 143 isolates (35.8%). Less frequently, *S. maltophilia* was detected in blood cultures (86 isolates, 21.6%), with the lowest number in urine samples (23/399, 5.8%).

Throughout the study period, the number of isolates from the lower respiratory tract ranged from 15 cases in 2020 to 32 cases in 2025, with a noticeable increase in 2023–2025 (28–30 isolates annually). A comparable trend was observed in wound- and skin-derived samples, rising from 27 cases in 2020 to a peak of 33 in 2024, and then decreasing to 26 in 2025.

Blood culture isolates remained relatively stable from 2020 to 2024, averaging 11–18 cases per year. However, a decline was observed in 2025, with 8 isolates recorded. Urine samples yielded consistently few isolates throughout the study period, averaging 1 to 6 cases annually and showing no marked year-to-year variability.

Table 1 summarizes the annual number of *S. maltophilia* isolates obtained from the four major categories of clinical materials—lower respiratory tract, blood, urine, and wound/skin samples—between 2020 and 2025.

Patients from whom *S. maltophilia* was isolated were hospitalized in a wide range of clinical departments. Across all years and material types, isolates most frequently originated from Hematology, Surgery, and related surgical specialties (including Plastic Surgery, Cardiac Surgery, and Orthopedics), as well as the COVID ward. Detailed departmental data are presented in Table 2.

Between 2020 and 2025, a total of 399 *Stenotrophomonas maltophilia* isolates were recovered from patients hospitalized across multiple clinical departments (Table 2). The median age of the patients was 68 years (IQR 56–76; range 17–95 years). Of these patients, 262 (65.7%) were male, and 137 (34.3%) were female. The highest cumulative number of isolates originated from the Intensive Care Unit (ICU; *n* = 94), followed by Hematology (*n* = 41), Plastic Surgery (*n* = 30), the Musculoskeletal Infections Unit (MIU; *n* = 27), Surgery (*n* = 24), and Cardiac Surgery (*n* = 23).

Bloodstream isolates accounted for 86 cases and were most frequently obtained from Hematology (*n* = 29) and the ICU (*n* = 17), followed by Cardiology (*n* = 15) and the COVID ward (*n* = 11). Fewer blood isolates were reported from Infectious Diseases (*n* = 3), Nephrology (*n* = 2), Surgery (*n* = 2), Neurosurgery (*n* = 1), Neurology (*n* = 1), and Vascular Surgery (*n* = 1). The highest annual number of bloodstream isolates was observed in 2023 and 2024 (*n* = 18 each).

Lower respiratory tract specimens yielded 143 isolates, predominantly associated with the ICU (*n* = 55), Pulmonology (*n* = 23), Cardiac Surgery (*n* = 11), Infectious Diseases (*n* = 8), Hematology (*n* = 5), and Neurosurgery (*n* = 5). Respiratory isolates were widely distributed across departments, with the annual total increasing from 14 in 2020 to 30 in 2025.

Urine-derived isolates were rare (*n* = 23) and occurred sporadically across departments, most commonly in Urology (*n* = 7), the ICU (*n* = 6), Nephrology (*n* = 4), and the COVID ward (*n* = 4). In most departments, urine isolates did not exceed one case per year.

Wound-, abscess-, and skin-derived specimens represented the largest group of isolates (*n* = 147). These were most frequently recovered from Plastic Surgery (*n* = 30), the MIU (*n* = 27), Surgery (*n* = 16), Vascular Surgery (*n* = 12), Cardiac Surgery (*n* = 6), and Hematology (*n* = 6). The highest annual number of wound-associated isolates was recorded in 2024 (*n* = 34), followed by 2023 (*n* = 30).

In summary, *S. maltophilia* was consistently recovered from high-intensity clinical departments, particularly in the ICU, hematology, and surgical specialties, over a six-year study period. The distribution of isolates across specimen types remained stable over the study period.

Antimicrobial susceptibility testing showed substantial year–to–year variation across the six-study period, with consistent patterns observed for trimethoprim–sulfamethoxazole (SXT), levofloxacin, and ceftazidime.

SXT retained high in vitro activity throughout the study period. Most isolates were categorized as susceptible with increased exposure (I) according to the EUCAST definitions, with no resistant isolates detected in 2020–2022. Low-level resistance appeared only sporadically thereafter, reaching 8% in 2023–2024 (six isolates) and 3% in 2025 (two isolates).

Levofloxacin susceptibility showed greater year-to-year variability than SXT during the period when EUCAST categorical interpretation was available. In 2020, 42% (25/60) of isolates were categorized as susceptible; none were in the susceptible, increased exposure (I) category, and 58% (35/60) were resistant. Susceptibility gradually increased in subsequent years, reaching 49% in 2022 and peaking at 65% in 2025. The proportion of isolates categorized as susceptible with increased exposure (I) rose from 0% in 2020 to 37% (28/76) in 2023. Resistance rates decreased markedly after 2020, remaining between 22% and 29% during 2022–2024, and then declining further to 14% (9/62) in 2025.

Overall, trimethoprim–sulfamethoxazole retained high activity against *S. maltophilia* isolates throughout the study period. Susceptibility results for levofloxacin and ceftazidime are presented descriptively for the years when EUCAST breakpoints were available, while categorical interpretation was discontinued following the 2024 EUCAST guidance.

A detailed summary of annual susceptibility profiles for SXT, levofloxacin, and ceftazidime is presented in Table 3. The distribution of *S. maltophilia* isolates across different clinical specimens from 2020 to 2025 is summarized in Table 4.

Ward-specific antibiotic consumption patterns are summarized in Table 5. Mean systemic antibacterial use ranged from 844.17 DDD/100 patient-days in 2020 to 886.05 in 2023 followed by a decline to 814.54 in 2025. Carbapenem consumption showed a similar trend, increasing from 75.10 DDD/100 patient-days in 2020 to 85.07 DDD/100 patient-days in 2023, and then decreasing to 69.34 DDD/100 patient-days in 2025. The highest antibiotic consumption was observed in the ICU, Cardiac Surgery, and the Musculoskeletal Infections Unit.

**Figure 1:**
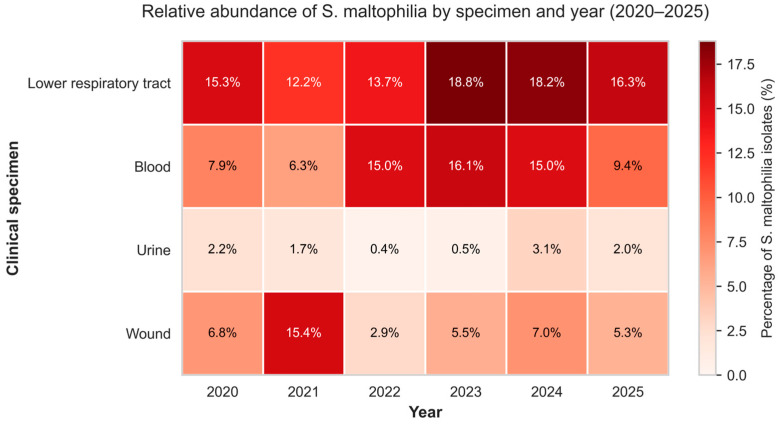
Heatmap of the relative abundance of *Stenotrophomonas maltophilia* by clinical specimen and year (2020–2025). Values represent the percentage of *S. maltophilia* isolates among all non-fermenting Gram-negative bacilli recovered from a given clinical specimen each year.

At the ward level, a moderate positive association was observed between mean carbapenem consumption and the total number of *S. maltophilia* isolates (Figure 1). Wards with higher carbapenem exposure generally exhibited a higher burden of *S. maltophilia*, although notable variability between wards was present.

A moderate positive correlation was observed between carbapenem consumption and isolate burden (r = 0.51, *p* = 0.043) (Figure 1). Given this moderate association, a multivariable linear regression analysis was performed to adjust for potential confounders.

This analysis confirmed that increased carbapenem pressure may contribute to the ecological selection of *S. maltophilia* within hospital wards. When a one-year temporal offset was introduced, the association with carbapenem consumption in the preceding year was stronger, suggesting a possible delayed ecological effect of antibiotic pressure on *S. maltophilia* isolation rates.

To investigate the ecological relationship between antimicrobial pressures and pathogen emergence, we analyzed the association between ward-level carbapenem consumption and *S. maltophilia* isolation burden across 16 clinical departments.

**Figure 2:**
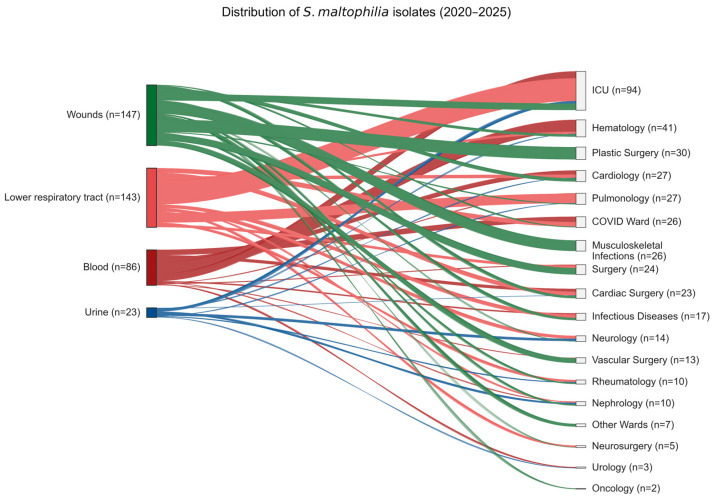
Specimen-to-ward distribution of *S. maltophilia* isolates (Sankey diagram, 2020–2025). The Sankey diagram visualizes the flow of *S. maltophilia* isolates from clinical specimen types (**left**) to hospital wards (**right**); ribbon width is proportional to the number of isolates. For each specimen type, the five wards with the highest isolate counts were identified. To improve readability, the diagram displays up to 12 wards with the highest overall counts among these candidates; all remaining wards were grouped as “Other wards”. Node labels show total isolate counts aggregated across 2020–2025.

Mean carbapenem consumption varied substantially across wards, ranging from 14.5 defined daily doses per 100 patient-days in Vascular Surgery to 237.9 in the Musculoskeletal Infection Unit (Figure 1). The highest carbapenem exposure was observed in the Musculoskeletal Infections Unit (237.9 DDD per 100 patient-days), Cardiac Surgery (196.9), Hematology (187.3), and the ICU (158.4). Similarly, the burden of *S. maltophilia* isolates was concentrated in high-intensity wards, with the ICU accounting for 94 isolates, followed by Hematology (41), Plastic Surgery (30), and the Musculoskeletal Infections Unit (27) (Figure 2).

At the ward level, a moderate positive correlation was observed between mean carbapenem consumption and the total number of *S. maltophilia* isolates (Pearson r = 0.51, *p* = 0.043), suggesting that wards with higher carbapenem exposure tended to exhibit a greater isolate burden. However, this unadjusted association did not account for potential confounding by ward type or overall antimicrobial pressure.

**Figure 3:**
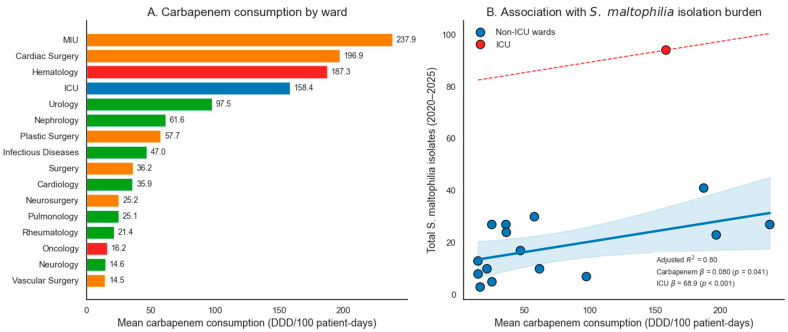
Ward-level carbapenem consumption and its association with *S. maltophilia* isolation burden. (**A**) displays the mean carbapenem consumption (expressed as defined daily doses per 100 patient-days, averaged across 2020–2025) across 16 clinical wards, arranged in descending order of consumption. Wards are color-coded by type: intensive care unit (blue), surgical specialties (orange), hematology/oncology (red), and medical wards (green). The Musculoskeletal Infections Unit (237.9 DDD/100 patient-days), Cardiac Surgery (196.9), Hematology (187.3), and the Intensive Care Unit (158.4) exhibited the highest carbapenem exposure. Numerical values are displayed at the end of each bar. (**B**) illustrates the association between mean carbapenem consumption (DDD/100 patient-days) and the total number of *S. maltophilia* isolates per ward over the six-year study period (2020–2025). Each point represents one clinical ward. The intensive care unit (ICU) is highlighted in red, while the non-intensive care wards are shown in blue. The solid blue line represents the predicted association for non-ICU wards derived from a multivariable linear regression model, with the shaded area indicating the 95% confidence interval. The dashed red line represents the parallel intercept shift for the ICU. In the adjusted model, both ICU status (β = 68.9, 95% CI 46.1–91.8, *p* < 0.001) and carbapenem consumption (β = 0.080 per DDD/100 patient-days, 95% CI 0.004–0.156, *p* = 0.041) were independently associated with increased *S. maltophilia* isolation burden (adjusted R^2^ = 0.80). DDD—defined daily dose; ICU—intensive care unit; MIU—Musculoskeletal Infections Unit.

To disentangle the independent contribution of carbapenem exposure and ward characteristics, we performed multivariable linear regression (Figure 3B; Table 6). After adjustment for ward type, ICU status emerged as the dominant predictor of *S. maltophilia* isolation burden (β = 68.9, 95% CI 46.1–91.8, *p* < 0.001), indicating that the ICU harbored approximately 69 additional isolates compared to the non-ICU ward, independent of antibiotic consumption patterns.

Importantly, carbapenem consumption remained independently associated with *S. maltophilia* isolation after adjustment for ICU status (β = 0.080 per 1 DDD/100 patient-days, 95% CI 0.004–0.156, *p* = 0.041; Table 6). This corresponds to approximately 0.8 additional isolates per 10-unit increase in carbapenem consumption. The overall model explained 80% of the variance in isolate counts (adjusted R^2^ = 0.80, F-statistic *p* < 0.001).

When total systemic antibiotic consumption (ATC J01) was included alongside carbapenem consumption in the multivariable model, neither variable achieved statistical significance, likely due to collinearity between these highly correlated predictors (Pearson r = 0.83, variance inflation factor > 5). This suggests that the observed ecological effect is attributable specifically to carbapenem pressure rather than to overall antimicrobial exposure.

To assess whether the association between carbapenem consumption and *S. maltophilia* isolation was driven primarily by intensive care settings, we repeated the analysis restricted to the 15 non-ICU wards. In this subset, carbapenem consumption remained significantly associated with isolate burden (β = 0.080, 95% CI 0.004–0.156, *p* = 0.041, R^2^ = 0.28), confirming that the ecological relationship between carbapenem pressure and *S. maltophilia* emergence extends beyond critical care environments to diverse clinical settings.

## 4. Discussion

In this six-year laboratory-based surveillance study, we analyzed the epidemiology and antimicrobial susceptibility of *Stenotrophomonas maltophilia* isolates recovered from hospitalized patients in a large tertiary care center. A total of 399 isolates were recovered between 2020 and 2025, representing a substantial sample size for a single-center European cohort. The distribution patterns of isolates across specimen types and clinical departments underscore the persistent presence and clinical relevance of *S. maltophilia* in high-intensity hospital environments [1,2].

The concentration of isolates in the ICU, hematology, and surgical wards mirrors the distribution patterns reported by previous studies [10]. These findings support the interpretation of *S. maltophilia* as a marker of severe underlying disease and complex clinical management rather than an environmentally insignificant contaminant. Although this retrospective study did not assess patient-level clinical outcomes, previous reports consistently describe high mortality associated with *S. maltophilia* infections, ranging from 21% to 69% [6], with crude mortality reaching 37–44% in ICU cohorts [11,12]. Independent risk factors for poor outcomes—such as septic shock, neutropenia, prolonged mechanical ventilation, and prior exposure to broad-spectrum antibiotics (especially carbapenems)—were typical of the patients treated in the wards with the highest isolation rates in our study.

The predominance of wound-, abscess-, and skin-derived isolates in our cohort is consistent with findings from other tertiary care hospitals, where *S. maltophilia* has increasingly been associated with surgical site infections and chronic wounds [13,14]. The organism’s ability to form biofilms on devitalized tissues and implanted devices likely facilitates its recovery in surgical patients, extending its clinical relevance beyond respiratory infections. Lower respiratory tract samples were the second most common source, showing a gradual increase over the study period. Similar trends have been reported in ICU-based studies, particularly among patients requiring prolonged mechanical ventilation [6,15]. In our dataset, however, detailed patient-level information regarding mechanical ventilation status was not systematically available, precluding assessment of its specific contribution to respiratory isolation patterns. Nevertheless, the predominance of respiratory isolates in high-intensity units supports the interpretation of *S. maltophilia* as an opportunistic pathogen thriving in environments characterized by invasive device use and sustained antimicrobial exposure. Bloodstream isolates accounted for 21.6% of cases, a proportion comparable to 10–25% rates reported in the literature [11,12], predominantly affecting immunocompromised patients. In contrast, urine-derived isolates remained rare, confirming *S. maltophilia* as an uncommon urinary pathogen usually associated with catheterization.

A key observation in our study was the increase in respiratory isolates after 2020, coinciding with the post–COVID-19 period. Several studies have documented a rise in opportunistic non-fermenting Gram-negative pathogens following the pandemic, linked to prolonged ICU stays and extensive antimicrobial exposure [16,17]. Our analysis of antibiotic consumption showed that carbapenem use peaked between 2021 and 2023, particularly in the ICU and surgical units. Given that *S. maltophilia* is intrinsically resistant to carbapenems, sustained exposure to these agents may create ecological niches that facilitate both its persistence and emergence. At the ward level, we observed a moderate positive association between mean carbapenem consumption and the total number of *S. maltophilia* isolates, indicating that hospital units with higher carbapenem exposure tended to exhibit a greater isolate burden. Although this ecological relationship does not establish causality, it supports the concept that sustained carbapenem pressure may facilitate the persistence and expansion of intrinsically resistant non-fermenting pathogens within the hospital environment. Interestingly, exploratory temporal analysis suggested that this association may exhibit a delayed pattern. This analysis was performed using aggregated annual ward-level data and did not allow reconstruction of individual patient-level exposure–isolation sequences. The relationship between carbapenem consumption and the number of *S. maltophilia* isolates was stronger than when antibiotic exposure in the preceding year was considered, suggesting that ecological dynamics of antimicrobial selection may be at play. Such a lag effect could be explained by the time required for antibiotic pressure to reshape microbial communities, facilitate environmental persistence, and ultimately increase the probability of clinical detection of intrinsically resistant organisms such as *S. maltophilia.* These observations suggest that *S. maltophilia* may serve as a potential ecological indicator of excessive carbapenem pressure in high-intensity hospital environments. Furthermore, hospital environmental reservoirs, such as contaminated water systems and plumbing biofilms, may have contributed to this persistence, as molecular studies (PFGE/MLST) have previously confirmed clonal transmission linked to these sources [2].

Our ward-level multivariable analysis provides quantitative evidence for an independent association between carbapenem consumption and *S. maltophilia* isolation burden, after adjustment for intensive care unit status. The observed effect size—approximately 0.8 additional isolates per 10-unit increase in carbapenem consumption—suggests a modest but clinically meaningful ecological relationship. Importantly, this association persisted when the analysis was restricted to non-intensive care wards, indicating that carbapenem stewardship is relevant across diverse clinical settings, not only in critical care environments.

The dominant effect of intensive care unit status in our multivariable model (β = 68.9, *p* < 0.001) reflects the cumulative impact of multiple risk factors that cluster in critical care settings, including patient severity, prolonged hospitalization, invasive device use, mechanical ventilation, and high antimicrobial exposure. The concentration of *S. maltophilia* isolates in intensive care units has been consistently reported in previous studies [1,2,6,10] and aligns with the organism’s ecology as an opportunistic pathogen that thrives in environments characterized by intense selective pressure and vulnerable patient populations [1,2].

It is important to emphasize that our analysis was conducted at the ward level using aggregated data, which precludes adjustment for individual patient characteristics such as age, comorbidities, severity of illness, and duration of antibiotic exposure. Ward-level associations reflect ecological patterns rather than individual risk, and the observed correlation may be confounded by differences in patient case-mix, infection control practices, or environmental contamination across wards. Nevertheless, ecological studies remain valuable for identifying system-level relationships between antimicrobial pressure and pathogen emergence, particularly in settings where patient-level data are unavailable [18,19].

Our findings provide hypothesis-generating evidence that warrants confirmation through prospective patient-level cohort studies with multivariable adjustment for clinical confounders.

The carbapenem effect was specific—it persisted even after accounting for total systemic antibiotic consumption—and is consistent with known resistance mechanisms. *S. maltophilia* possesses intrinsic resistance to carbapenems mediated by constitutive expression of L1 and L2 metallo-β-lactamases, efflux pumps, and low outer membrane permeability [2,7]. Sustained carbapenem exposure creates an ecological niche by suppressing carbapenem-susceptible competitors (including *Enterobacterales* and other Gram-negative pathogens), thereby facilitating the selection and persistence of intrinsically resistant organisms such as *S. maltophilia*. Our findings align with the concept of collateral selection pressure, whereby the use of broad-spectrum antibiotics promotes the emergence of intrinsically resistant organisms. The ecological dynamics have been documented across multiple healthcare settings and underscore the importance of targeted carbapenem stewardship to mitigate the emergence of non-fermenting Gram-negative pathogens [20,21].

From a preventive perspective, our findings underscore the need for sustained and targeted carbapenem stewardship, particularly in high-intensity wards. Early reassessment and de-escalation of broad-spectrum antimicrobial therapy, when clinically feasible, may help reduce ecological selection pressure favoring intrinsically resistant organisms. In addition, strict adherence to infection prevention and control measures—including hand hygiene compliance and appropriate management of invasive medical devices—is essential to limit both selection and potential transmission. Although routine environmental screening is not indicated outside outbreak settings, vigilance regarding water-associated reservoirs remains important in the context of unexplained clustering. Continuous microbiological surveillance at the ward level may further support early detection of emerging trends and guide preventive interventions.

Antimicrobial susceptibility testing demonstrated sustained activity of trimethoprim–sulfamethoxazole (SXT), consistent with its role as the first-line treatment [2]. However, the predominance of isolates categorized as “susceptible with increased exposure” (I) reflects the impact of the revised EUCAST breakpoints and underscores the need for optimized dosing strategies. Levofloxacin susceptibility showed marked interannual variability across European cohorts, while ceftazidime retained good in vitro activity prior to the 2024 EUCAST breakpoint withdrawal [22].

Despite its documented clinical relevance, *S. maltophilia* is currently not included in the WHO Global Antimicrobial Resistance and Use Surveillance System (GLASS) [23]. This surveillance gap contributes to an underestimation of its true burden. While *S. maltophilia* remains less prevalent than *Pseudomonas aeruginosa* or *Acinetobacter baumannii*, its persistent presence across specimen types and high-risk wards challenges the perception of this organism as a marginal pathogen [1].

Although certain wards, such as the Musculoskeletal Infections Unit, showed repeated isolation of *S. maltophilia* in selected years (e.g., nine wound isolates in 2020 and seven in 2024), these cases were distributed throughout the respective calendar years and were not recognized as temporally clustered outbreaks by the hospital infection prevention and control team. The hospital operates under established Infection Prevention and Control policies in accordance with national and European guidelines, including routine surveillance of multidrug-resistant organisms, standard and transmission-based precautions, environmental hygiene protocols, and an active antimicrobial stewardship program. The temporal distribution of cases across calendar years suggests endemic persistence rather than outbreak dynamics. However, molecular typing was not performed in this study; therefore, it was not possible to determine whether isolates represented clonal transmission or multiple unrelated strains. Systemic environmental sampling was not performed during the study period and is routinely undertaken at our institution only during suspected outbreak investigations. As this analysis was conducted at the ward level using aggregated surveillance data, conclusions regarding transmission dynamics must be interpreted with caution. Consequently, while no formally declared outbreak occurred during the study period, silent clonal spread cannot be definitively excluded.

This study has several limitations. First, the retrospective design precluded reliable differentiation between colonization and infection, potentially leading to an overestimation of the clinical burden. Second, patient-level clinical data (e.g., immunosuppressive status, corticosteroid therapy, chemotherapy, diabetes mellitus, invasive device use including central venous catheters, mechanical ventilation, severity of illness, and mortality outcomes) were not available, limiting our ability to assess clinical outcomes. Third, this study was designed as an ecological analysis at the ward level, using aggregated antibiotic consumption and isolate counts. The observed association between carbapenem consumption and *S. maltophilia* isolation does not establish causality, as individual patient-level data (e.g., age, comorbidities, illness severity, duration of antibiotic exposure, invasive device use) were unavailable for multivariable adjustment. Unmeasured confounders, including patient case-mix, environmental contamination, and infection control practices, may have influenced this association. Ward-level associations reflect ecological patterns rather than individual risk, and the observed correlation may be confounded by differences in patient characteristics across wards. These findings should be interpreted as ecological associations and do not establish causality at the individual patient level. Nevertheless, ecological studies remain valuable for identifying system-level relationships between antimicrobial pressure and pathogen emergence, particularly in settings where patient-level data are unavailable [18,19]. Our findings provide hypothesis-generating evidence that warrants confirmation through prospective patient-level cohort studies with multivariable adjustment for clinical confounders. Fourth, susceptibility testing was not performed uniformly across all years due to changes in laboratory protocols and EUCAST guidance, potentially affecting temporal comparisons. Nevertheless, the extended six-year observation period and the large number of isolates provide a robust foundation for identifying early indicators of emerging trends, informing empirical therapy, and supporting future efforts to include clinically relevant non-fermenters in international monitoring. Finally, the single-center design may limit generalizability to other healthcare settings.

## 5. Conclusions

This six-year study demonstrates that *Stenotrophomonas maltophilia* is a persistent and clinically relevant opportunistic pathogen in tertiary care settings. Its consistent prevalence in intensive care units, hematology, and surgical wards suggests that its isolation reflects patient severity and healthcare complexity rather than incidental environmental contamination. The post-pandemic increase in respiratory isolates further underscores the dynamic nature of its hospital epidemiology, likely driven by shifting antimicrobial pressures.

From a clinical perspective, our findings reinforce trimethoprim–sulfamethoxazole as the cornerstone of empirical therapy, while the emergence of the ‘susceptible, increased exposure’ (I) category necessitates optimized, high-dose regimens to ensure clinical efficacy. Furthermore, the correlation between carbapenem consumption and *S. maltophilia* isolation highlights the critical need for restrictive carbapenem stewardship to limit the expansion of this intrinsically resistant pathogen. In high-risk wards, the recovery of *S. maltophilia* may represent an important clinical signal requiring prompt evaluation of the patient’s infection status.

Given that *S. maltophilia* is not currently included in the WHO Global Antimicrobial Resistance and Use Surveillance System (GLASS), our findings provide critical evidence of its clinical burden. These results advocate for the integration of *S. maltophilia* into local and international antimicrobial monitoring frameworks and its inclusion in routine infection prevention and stewardship strategies.

## Figures and Tables

**Table 1:** Annual distribution of *Stenotrophomonas maltophilia* isolates by biological material (2020–2025).

Biological Material	Year						Total
	2020	2021	2022	2023	2024	2025	
Blood	11	14	17	18	18	8	86
Lower respiratory tract	15	22	18	28	28	32	143
Urine	6	4	1	1	6	5	23
Wounds, abscesses, skin lesions	27	28	13	28	33	26	147

Values represent the number of non-duplicate *S. maltophilia* isolates recovered each year from individual specimen types. The last column presents the total number of isolates obtained from each biological material over the six–year study period.

**Table 2:** Distribution of *S. maltophilia* isolates across hospital departments by year, 2020–2025.

Source of Biological Material	Years	ICU	Hematology	Cardiology	Rheumatology	Nephrology	Pulmonology	Surgery	Plastic Surgery	Cardiac Surgery	Vascular Surgery	Neurosurgery	Infectious Diseases	Neurology	Urology	Oncology	MIU	COVID Ward	Others	Total
Blood	2020	0	3	5	0	1	0	1	0	0	0	0	2	0	0	0	0	0	0	12
	2021	1	1	0	0	0	0	0	0	5	0	0	0	0	0	0	0	7	0	14
	2022	1	6	3	0	0	1	0	0	2	0	0	0	0	0	0	0	4	0	17
	2023	9	5	0	0	0	0	0	0	0	0	0	0	0	0	2	0	0	2	18
	2024	4	10	2	0	0	0	1	0	0	0	0	1	0	0	0	0	0	0	18
	2025	2	4	0	0	0	0	0	0	0	1	0	0	0	0	0	0	0	0	7
Lower respiratory tract	2020	3	1	2	1	0	3	0	0	3	0	1	0	0	0	0	0	0	0	14
	2021	5	0	0	0	0	0	2	0	2	0	0	5	0	0	0	0	10	0	24
	2022	8	0	0	0	1	5	0	0	1	0	2	1	2	0	0	0	1	0	21
	2023	12	2	3	2	0	6	0	0	0	0	0	1	1	0	0	0	0	0	27
	2024	13	1	0	1	1	4	3	0	1	0	0	1	2	0	0	0	0	0	27
	2025	14	1	3	1	0	5	1	0	2	0	1	0	2	0	0	0	0	0	30
Urine	2020	1	0	0	1	1	0	0	0	1	0	0	0	0	1	0	0	0	0	5
	2021	1	1	0	0	1	0	0	0	0	0	0	0	0	2	0	0	0	0	5
	2022	1	0	0	0	0	0	0	0	0	0	0	0	0	1	0	0	0	0	2
	2023	0	0	0	0	0	0	0	0	0	0	0	0	0	1	0	0	0	0	1
	2024	1	0	1	0	1	1	0	0	0	0	0	0	0	2	0	0	0	0	6
	2025	2	0	0	1	1	0	0	0	0	0	0	0	0	0	0	0	0	0	4
Wounds, abscesses, skin lesions	2020	0	1	2	1	1	0	7	2	1	2	0	0	0	0	0	9	0	0	26
	2021	2	2	1	1	0	0	1	0	0	3	1	3	0	0	0	2	0	2	18
	2022	2	0	0	0	0	0	2	2	0	0	0	1	0	0	1	4	0	2	14
	2023	4	1	0	0	1	2	1	15	2	1	0	0	0	0	0	1	0	2	30
	2024	4	2	3	1	1	0	1	7	2	3	0	1	1	0	0	7	0	1	34
	2025	4	0	2	0	0	0	4	4	1	3	0	1	0	0	0	4	0	2	25
total		94	41	27	10	10	27	24	30	23	13	5	17	8	7	3	27	22	11	399

Values represent the annual number of *S. maltophilia* isolates recovered from each hospital department. Data include all clinical samples (blood, lower respiratory tract, urine, and wound swab). The last row summarizes the total number of isolates per department for the entire study period. MIU—Musculoskeletal Infections Unit.

**Table 3:** Antimicrobial susceptibility of *Stenotrophomonas maltophilia* isolates to trimethoprim–sulfamethoxazole, levofloxacin, and ceftazidime (2020–2025).

Year	Number of Isolates	SXT			LEV			CAZ		
		*n* (%S)	*n* (%I)	*n* (%R)	*n* (%S)	*n* (%I)	*n* (%R)	*n* (%S)	*n* (%I)	*n* (%R)
2020	60	0	60 (100%)	0	25 (42%)	0	35 (58%)	22 (37%)	0	38 (63%)
2021	61	0	61 (100%)	0	25 (41%)	19 (31%)	17 (28%)	19 (31%)	0	42 (69%)
2022	51	0	51 (100%)	0	25 (49%)	15 (29%)	11 (22%)	9 (18%)	0	42 (82%)
2023	76	0	70 (92%)	6 (8%)	n/a	n/a	n/a	n/a	n/a	n/a
2024	79	0	73 (92%)	6 (8%)	n/a	n/a	n/a	n/a	n/a	n/a
2025	62	0	60 (97%)	2 (3%)	n/a	n/a	n/a	n/a	n/a	n/a

Values represent the number and percentage of isolates classified as susceptible (S), susceptible increased exposure (I), or resistant (R) based on yearly antimicrobial susceptibility testing. Percentages shown in parentheses are calculated relative to the total number of isolates tested each year. SXT—trimethoprim–sulfamethoxazole; LEV—levofloxacin; CAZ—ceftazidime; n/a—not applicable.

**Table 4:** Number of *Stenotrophomonas maltophilia* isolates in comparison with other non-fermenting Gram-negative bacilli by clinical specimen (2020–2025).

Year	Material	*S. maltophilia* *n*/N (%)	*P. aeruginosa* *n*/N (%)	*A. baumannii* *n*/N (%)
2020	lower respiratory tract	15/98 (15.3%)	48/98 (49.0%)	35/98 (35.7%)
	blood	11/139 (7.9%)	61/139 (43.9%)	67/139 (48.2%)
	urine	6/272 (2.2%)	168/272 (61.8%)	98/272 (36.0%)
	wounds	27/396 (6.8%)	235/396 (59.3%)	134/396 (33.8%)
2021	lower respiratory tract	22/181 (12.2%)	47/181 (26.0%)	112/181 (61.9%)
	blood	14/223 (6.3%)	74/223 (33.2%)	135/223 (60.5%)
	urine	4/234 (1.7%)	119/234 (50.9%)	111/234 (47.3%)
	wound	20/130 (15.4%)	87/130 (66.9%)	23/130 (17.7%)
2022	lower respiratory tract	18/131 (13.7%)	54/131 (41.2%)	59/131 (45.0%)
	blood	17/113 (15.0%)	52/113 (46.0%)	44/113 (38.9%)
	urine	1/237 (0.4%)	153/237 (64.6%)	83/237 (35.0%)
	wound	13/456 (2.9%)	246/456 (53.9%)	197/456 (43.2%)
2023	lower respiratory tract	28/149 (18.8%)	63/149 (42.3%)	58/149 (38.2%)
	blood	18/111 (16.2%)	31/111 (27.9%)	62/111 (55.9%)
	urine	1/203 (0.5%)	110/203 (54.2%)	92/203 (45.3%)
	wound	28/508 (5.5%)	299/508 (58.9%)	181/508 (36.0%)
2024	lower respiratory tract	28/154 (18.2%)	79/154 (51.3%)	47/154 (30.5%)
	blood	18/119 (15.1%)	64/119 (53.8%)	32/119 (26.9%)
	urine	6/193 (3.1%)	137/193 (71.0%)	50/193 (25.9%)
	wound	33/470 (7.0%)	313/470 (66.6%)	124/470 (26.4%)
2025	lower respiratory tract	32/196 (16.3%)	101/196 (51.5%)	63/196 (32.1%)
	blood	8/85 (9.4%)	44/85 (51.8%)	33/85 (38.8%)
	urine	5/250 (2.0%)	142/250 (56.8%)	103/250 (41.2%)
	wound	26/487 (5.3%)	288/487 (59.1%)	173/487 (35.5%)

The table presents the number of isolates of a given pathogen obtained from a specific biological material (*n*), in relation to the total number of non-fermenting Gram-negative bacilli isolates recovered from the same biological material (N), along with the corresponding percentage (*n*/N).

**Table 5:** Antibiotic consumption expressed as DDD per 100 patient-days (ATC J01) and carbapenem use (J01DH) across hospital wards, 2020–2025.

Ward	Antibacterials for Systemic Use (J01)						Carbapenems (J01DH)					
	2020	2021	2022	2023	2024	2025	2020	2021	2022	2023	2024	2025
Haematology	692.60	730.90	817.25	884.11	889.10	863.28	133.52	187.78	192.13	203.87	237.61	168.81
ICU	1198.89	1966.76	2180.40	2389.54	1317.97	1505.29	185.01	167.20	168.36	196.89	137.53	94.53
Cardiology	823.44	519.38	822.48	754.42	755.41	870.91	43.79	36.17	41.84	31.11	24.81	37.90
Rheumatology	898.23	625.89	514.82	481.05	497.39	573.59	23.12	25.14	20.46	18.10	19.84	22.48
Nephrology	802.34	678.63	765.27	732.41	715.67	725.21	76.16	55.17	58.04	68.38	60.19	51.83
Pulmonology	1118.32	903.33	869.70	869.12	1012.01	895.86	21.43	19.96	29.61	29.63	24.59	25.60
Surgery	691.97	674.72	594.97	559.01	548.52	481.18	48.48	47.13	33.03	37.48	29.79	21.51
Plastic Surgery	953.32	919.52	1011.80	1053.38	1089.55	1012.09	87.67	53.55	74.07	62.73	25.55	42.54
Cardiac Surgery	1530.15	1553.85	1683.21	1623.14	1571.00	1604.65	178.19	159.12	195.13	177.27	207.60	264.15
Neurosurgery	392.61	413.70	355.48	417.63	330.74	184.54	37.77	31.23	19.86	40.82	14.82	7.08
Vascular Surgery	504.31	563.94	400.67	313.63	415.06	410.34	18.32	13.27	10.53	13.31	21.49	10.16
Infectious Diseases	898.08	885.50	691.66	833.42	785.93	808.79	27.24	50.36	43.41	57.97	54.38	48.26
Neurology	491.59	362.51	405.39	429.25	409.54	377.47	32.16	12.43	12.73	16.22	13.91	10.10
Urology	890.75	845.74	799.29	907.95	857.61	881.97	87.96	86.76	93.14	124.06	100.00	93.22
Oncology	126.31	180.68	139.90	210.83	110.32	108.43	7.34	20.74	11.06	23.07	17.48	16.24
MIU	1493.82	1386.87	1631.77	1717.98	1540.51	1728.96	193.48	258.15	244.31	260.18	259.89	195.06
Mean	844.17	825.75	855.25	886.05	802.90	814.54	75.10	76.51	77.98	85.07	78.09	69.34

All values are expressed as defined daily doses (DDD) per 100 patient-days, according to the WHO Anatomical Therapeutic Chemical (ATC) classification system.

**Table 6:** Association between ward-level factors and *S. maltophilia* isolation burden: multivariable linear regression results.

Factor	Additional Isolates Associated with Factor	95% Confidence Interval	*p*-Value
Intensive care unit ward (versus non-ICU)	69	46–92	<0.001
Carbapenem consumption (+10 DDD/100 patient-days) ^a^	0.8	0.04–1.56	0.041

Model summary: the model explained 80% of the variance in *S. maltophilia* isolation burden across wards (adjusted R^2^ = 0.80, *p* < 0.001). ^a^ For every 10-unit increase in mean carbapenem consumption (defined daily doses per 100 patient-days), the expected number of *S. maltophilia* isolates increased by approximately 0.8, after adjusting for ward type. Interpretation: the intensive care ward had approximately 69 more *S. maltophilia* isolates than the non-intensive care wards over the six-year study period, independent of carbapenem consumption. Additionally, higher carbapenem consumption was independently associated with greater isolation burden, with each 10-unit increase in consumption linked to approximately one additional isolate. DDD—defined daily dose; ICU—intensive care unit.

## Data Availability

The data from this study are available on request from the corresponding author.

## Abbreviations

The following abbreviations are used in this manuscript:

AMR	Antimicrobial Resistance
ATC	Anatomical Therapeutical Chemical classification
CAZ	Ceftazidime
DDD	Defined Daily Dose
EUCAST	European Committee on Antimicrobial Susceptibility Testing
GLASS	Global Antimicrobial Resistance and Use Surveillance System
ICU	Intensive Care Unit
LEV	Levofloxacin
MALDI-TOF MS	Matrix-Assisted Laser Desorption/Ionization Time-of-Flight Mass Spectrometry
MIU	Musculoskeletal Infection Unit
SXT	Trimethoprim–sulfamethoxazole
WHO	World Health Organization
